# Long-term mortality in pediatric sepsis: a systematic review and meta-analysis

**DOI:** 10.1080/07853890.2026.2617403

**Published:** 2026-01-19

**Authors:** Yongbiao Lv, Jiayi Zheng, Junxiang Cai, Jingwei Shui, Yuntao Liu, Zhongde Zhang

**Affiliations:** a The Second School of Clinical Medicine, Guangzhou University of Chinese Medicine, Guangzhou, Guangdong, China; b The First School of Clinical Medicine, Guangzhou University of Chinese Medicine, Guangzhou, Guangdong, China; c The Second Affiliated Hospital of Guangzhou University of Chinese Medicine, Guangzhou, Guangdong, China

**Keywords:** Long term, meta-analysis, mortality, pediatric sepsis

## Abstract

**Background:**

Pediatric sepsis represents a significant factor in the mortality rates among children, with survivors remaining highly fragile during the period following discharge. While in-hospital and short-term mortality have been widely studied, the long-term mortality of pediatric sepsis is not adequately synthesized or appreciated. This study aims to estimate the long-term mortality associated with pediatric sepsis, providing a basis for optimizing post-discharge surveillance and care protocols.

**Methods:**

This systematic review and meta-analysis followed PRISMA guidelines and was registered in PROSPERO (CRD420251137504). Exhaustive searches were conducted in PubMed, Embase, the Cochrane Library, and Web of Science for studies published from the inception of each database to June 30, 2025. Studies reporting long-term mortality in pediatric sepsis patients diagnosed using international consensus criteria were included. After literature screening, long-term mortality was pooled using a random effects meta-analysis in R statistical software.

**Results:**

A total of 72,065 records were identified through database searching. After removing duplicates and screening, six studies comprising 11,318 pediatric sepsis patients were included. The pooled long-term mortality in pediatric sepsis was 11% (95% CI: 7–16%), though significant heterogeneity was observed (*I*^2^ = 98.2%, *p* < 0.001). Sensitivity analyses yielded similar results, and evidence of publication bias was limited.

**Conclusion:**

Long-term mortality after pediatric sepsis was 11%, highlighting the persistent risk of mortality after hospital discharge. Further high-quality longitudinal studies are required to identify modifiable risk factors and guide evidence-based follow-up and personalized care.

## KEY MESSAGES

- This first meta-analysis on the long-term mortality in pediatric sepsis survivors reveals that the pooled long-term mortality after pediatric sepsis is 11%.
- Pediatric sepsis survivors remain highly vulnerable after discharge, with their post-discharge mortality being a severely underestimated issue that stays high regardless of the follow-up period or settings.

## Background

Pediatric sepsis is defined as a Phoenix Sepsis Score of at least 2 points with suspected or confirmed infection in children under 18 years, excluding neonates or newborns with a postconceptional age younger than 37 weeks [1]. In 2017, global estimates reported that approximately 25 million children experienced sepsis, resulting in over 3 million fatalities [2]. Recent studies indicate a consistent decline in both the incidence and mortality of sepsis among individuals younger than 14 years from 1990 to 2021 [3]. Specifically, the number of post-neonatal infant cases decreased from 27.1 million in 1990 to 13.3 million in 2021, and the proportion of sepsis-related deaths among neonates declined from 53.9 to 44.4% of all deaths during the same period [3]. The median hospitalization cost per pediatric sepsis case was $26,592, with annual expenditures reaching $7.31 billion, accounting for 18.1% of national pediatric hospitalization costs in 2019 [4]. Despite the case-fatality rates of pediatric severe sepsis and septic shock having declined over time, the fatality rate was higher in the developing countries than in the developed countries during the same period [5]. Nevertheless, many pediatric sepsis survivors demonstrate persistent sequelae clinically manifested by elevated post-discharge healthcare resource utilization [6].

The expanding cohort of pediatric sepsis survivors has engendered heightened clinical focus on their multidimensional long-term health trajectories, particularly encompassing physical, cognitive, and emotional health [7]. A cohort study of critically ill children revealed that 20% of sepsis survivors manifested new-onset or exacerbation of target conditions [8]. In one systematic review, pediatric sepsis survivors are more at risk for cognitive delay, visual impairment, hearing impairment, and cerebral palsy [9]. In addition, the 28-day mortality rate in children with septic shock is reaching 67%, and health-related quality of life decline by greater than 10% occurred in 31.0% children surviving septic shock [10].

Sustainable Development Goal 3.2 seeks to eradicate preventable fatalities of newborns and children under 5 years of age by 2030 [11]. The vast majority of research on pediatric sepsis looked at either 28-day mortality or in-hospital mortality, while the long-term mortality remains unappreciated [12]. However, post-discharge mortality accounted for 50.8% of all pediatric sepsis-related fatalities, with 7.9% of sepsis survivors in the 0–6 month age cohort experiencing mortality following hospital discharge [13]. These finding underscores that pediatric sepsis survivors remain highly vulnerable during the post-discharge period.

Despite emerging studies on long-term mortality after pediatric sepsis, the absence of comprehensive systematic reviews has resulted in fragmented evidence and inadequate synthesis. The purpose of this systematic review and meta-analysis is to estimate the long-term mortality risks associated with pediatric sepsis, providing a basis for optimizing post-discharge surveillance and care protocols.

## Methods

This review was conducted following the Preferred Reporting Items for Systematic Reviews and Meta-Analyses (PRISMA) guidelines[14] (Supplementary Table 1). The study protocol was registered in PROSPERO (CRD420251137504).

## Data sources and search strategy

We systematically searched PubMed, Embase, the Cochrane Library, and Web of Science for studies published from the inception of each database to 30 June, 2025. The search combined Medical Subject Headings (MeSH) and free-text terms using Boolean operators, including terms such as ‘sepsis’, ‘child’, ‘mortality’, and ‘follow-up study’. The full electronic search strategy for each database is provided in Supplementary Table 2.

## Inclusion and exclusion criteria

Eligible studies fulfilled all the following criteria: (1) Population: pediatric sepsis patients diagnosed using international consensus definitions [1,15]. (2) Outcome: reporting long-term mortality, defined as mortality at least 30 days after sepsis diagnosis or hospital discharge. (3) Study design: cohort, cross-sectional, or randomized controlled trials in humans.

Studies were excluded if they met all of the following criteria: (1) Focus on adults (≥18 years), preterm neonates (<37 weeks’ gestation at birth), or mixed populations without pediatric-specific data. (2) Lack of extractable data on long-term mortality. (3) Were case reports, reviews, editorials, comments, letters, biographies, books, news items, conference abstracts, expressions of concern, retractions, or animal studies. (4) Duplicate or overlapping reports from the same cohort. (5) Not published in English.

## Selection process

After deduplication using EndNote X9, two independent reviewers (YL and JZ) screened titles/abstracts and subsequently assessed full texts for eligibility. Discrepancies were resolved through consultation with a third reviewer (JS).

## Data extraction

From each included study, we extracted the following data: first author, publication year, country, population characteristics, study period, research period, follow-up duration, loss-to-follow-up rate, mortality, and data source.

## Quality assessment

Study quality was independently evaluated by two reviewers (YL and JZ) using the Newcastle-Ottawa scale (NOS) [16]. Disagreements were adjudicated by a third reviewer (JS). The NOS assessed three domains: selection (0–4 points), comparability (0–2 points), and outcome assessment (0–3 points). For studies lacking comparison groups, a modified NOS (0–6 points) was applied. A NOS score <4 was considered indicative of high risk of bias.

## Statistical analyses

All statistical analyses were performed in R (version 4.4.2). Pooled long-term mortality rates were estimated using a meta-analysis of proportions with the meta package [17]. Proportions were transformed using the Freeman-Tukey double arcsine transformation to stabilize variances and improve normality. Heterogeneity was evaluated using Cochran’s *Q* and *I*^2^ statistics [18]. Given anticipated heterogeneity, pooled estimates were calculated using a random-effects model.

Results are presented as forest plots showing individual study estimates with 95% confidence intervals (CI) and study weights. To minimize the excessive influence of individual studies on the pooled effect size, sensitivity analysis was conducted by iteratively excluding each study to assess its impact on the pooled estimate. Subgroup analyses were conducted to assess robustness and explore sources of heterogeneity. Publication bias was evaluated by inspection of funnel plot symmetry and quantified with Egger’s test [19].

## Results

### Study selection

**Figure 1.**
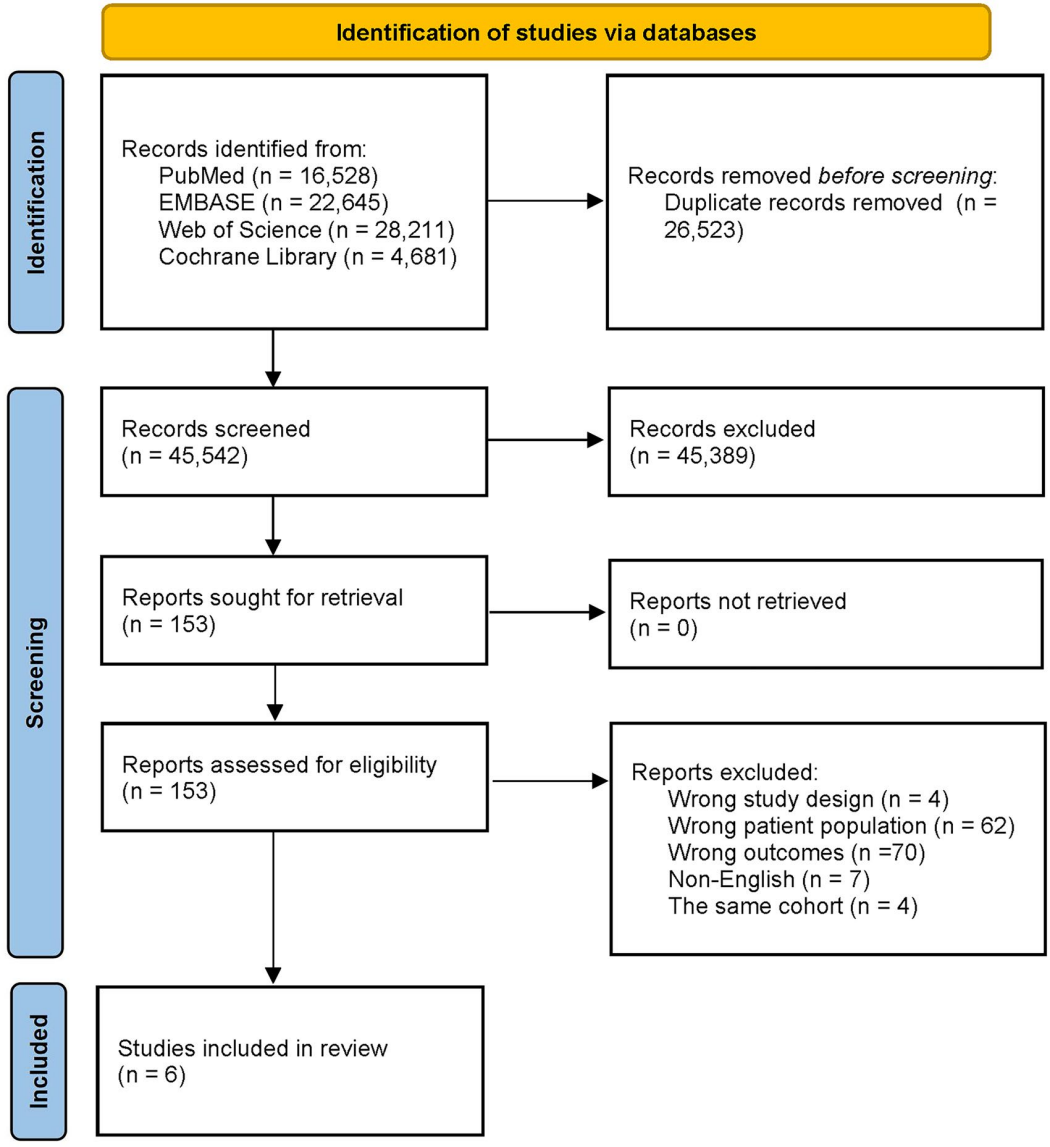
PRISMA flow diagram of study selection.

A total of 72,065 records were identified through database searching. After removing 26,676 duplicates, 45,389 articles were excluded based on title and abstract screening. Following full-text review, six studies met eligibility criteria and were included in the meta-analysis. The detailed screening process is shown in Figure 1.

### Study characteristics

The meta-analysis included six studies comprising 11,318 pediatric sepsis patients from diverse regions. Among the included studies, Nazir et al. were the first to prospectively collect 60-day mortality data in children with septic shock in India [20]. Zimmerman et al. conducted a 12-month follow-up multicenter study in the United States between 2013 and 2017 to investigate the long-term mortality trajectories in this population [21]. Wösten-van Asperen and colleagues reported 90-day mortality among pediatric cancer patients with sepsis in a multicenter retrospective cohort study across Europe and the Americas [22]. Wiens et al. performed the largest multicenter prospective study in Uganda involving children under five years of age with suspected sepsis and reported six-month mortality rates [23]. In contrast, the studies by He et al. and Chen et al. were both retrospective studies conducted in China with relatively small sample sizes [24,25]. Key characteristics are summarized in Table 1.

**Table 1.** Characteristics of included studies.

Author/year	Country	Study design	Study period	Multicenter	Sample size	Mortality
Zimmerman J J et al. 2020 [21]	USA	Prospective	2014–2017	Yes	389	1, 3, 6, and 12 Months
Wösten-van Asperen R M et al. 2023 [22]	Multinational	Retrospective	2012–2020	Yes	2281	1, 2, 3 Months
Nazir M et al. 2019 [20]	India	Prospective	2015–2016	No	83	2 Months
Wiens M O et al. 2024 [23]	Uganda	Prospective	2012–2021	Yes	8340	6 Months
He M et al. 2024 [25]	China	Retrospective	2020–2024	No	150	3 Months
Chen J et al. 2024 [24]	China	Retrospective	2015–2021	No	75	2 months

### Quality evaluation

Overall study quality was acceptable, with Newcastle-Ottawa Scale (NOS) scores ranging from 4 to 6 (Supplementary Table 3). All studies lost points in the comparability domain and in the selection of a non-exposed cohort because of the absence of suitable control groups. Additionally, certain studies received lower scores due to limited generalizability from highly specific populations and follow-up rates of less than 90%.

### Long-term mortality

**Figure 2.**
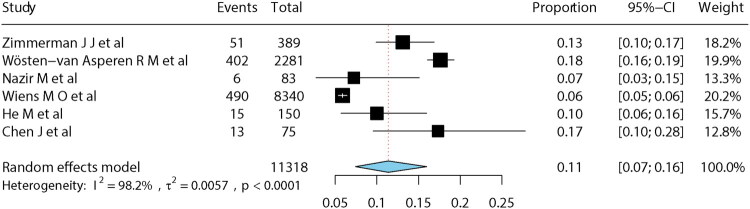
Forest plot of pooled long-term mortality in pediatric sepsis.

For the meta-analysis, we used each study’s longest reported follow-up time point. The pooled long-term mortality among pediatric sepsis patients was 11% (95% CI: 7–16%). Substantial heterogeneity was present (*I*^2^ = 98.2%, *p* < 0.001) (Figure 2).

### Sensitivity analysis and publication bias

**Figure 3.**
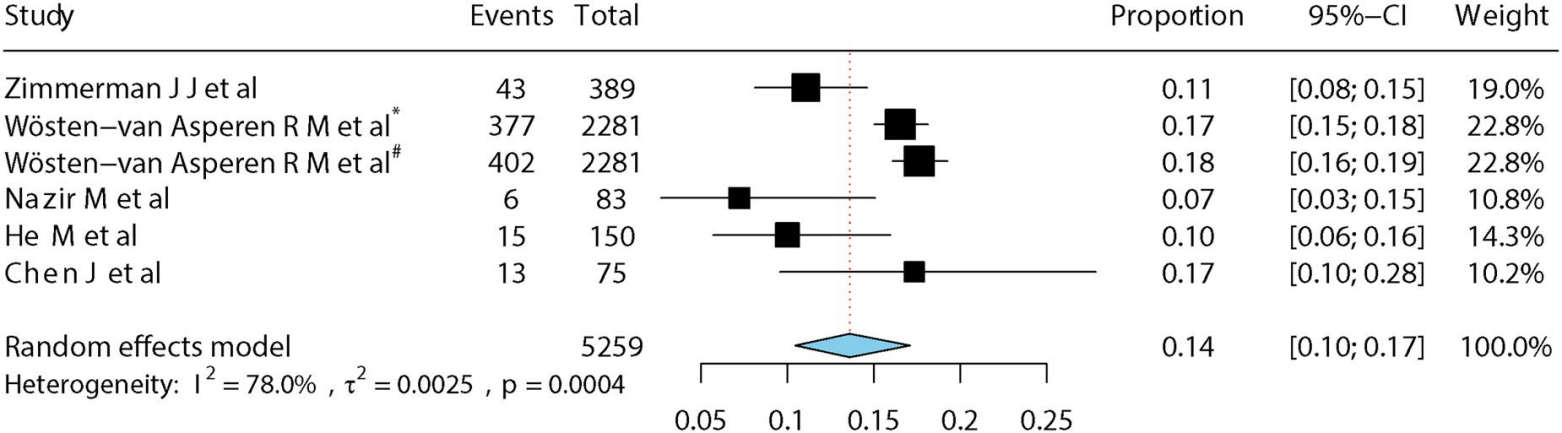
Mortality at 1–3 months. *Note*: *Mortality in 2 months, ^#^mortality in 3 months

**Figure 4.**
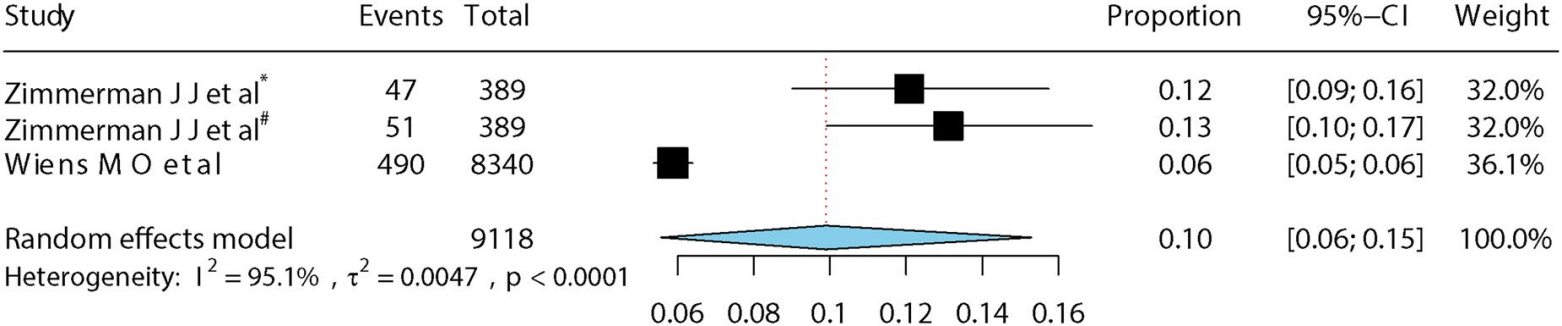
Mortality at 6–12 months. *Note*: *Mortality in 6 months, ^#^mortality in 12 months.

**Figure 5.**
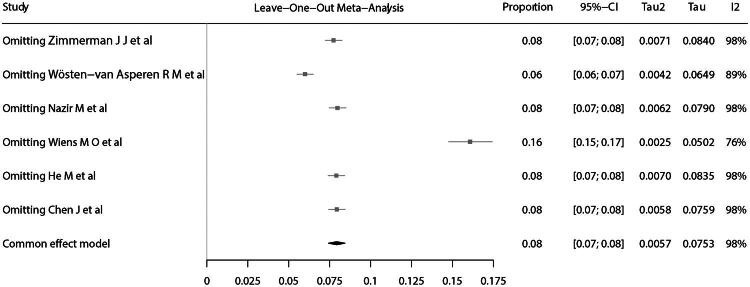
Leave-one-out sensitivity analysis.

**Figure 6.**
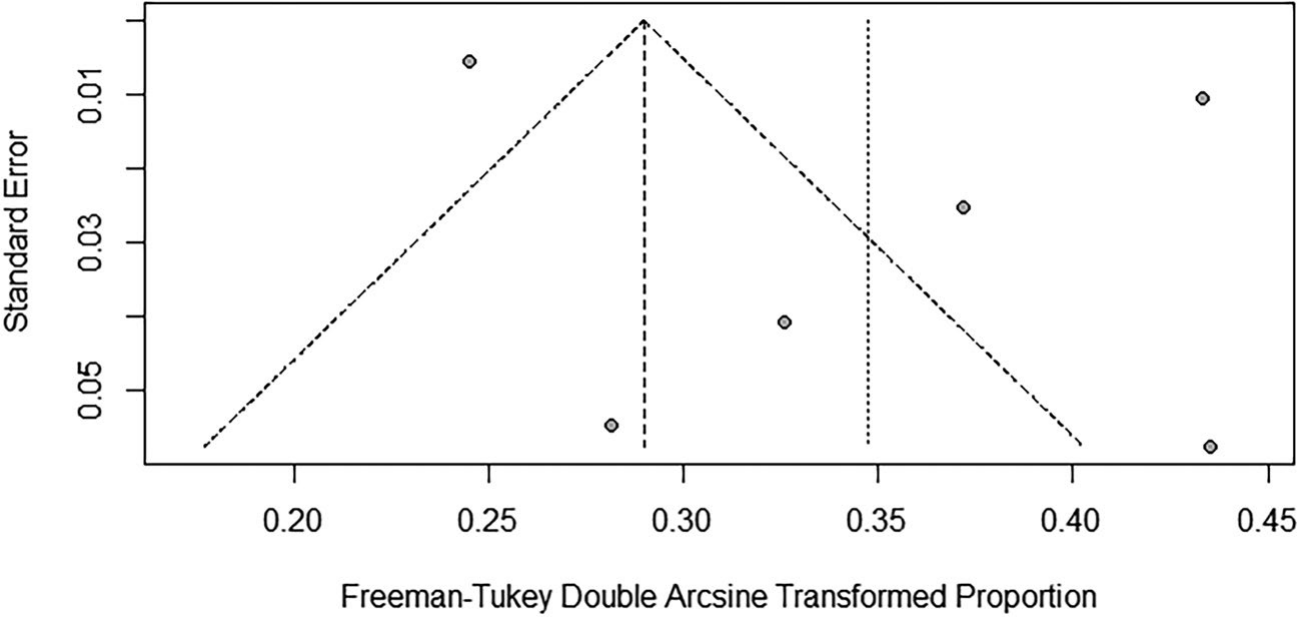
Funnel plot.

Because follow-up duration varied across studies, subgroup analyses were performed for studies with follow-up ≤3 months and those with follow-up of 6–12 months. The pooled long-term mortality was 14% (95% CI: 10–17%) for follow-up ≤3 months and 10% (95% CI: 6–15%) for follow-up of 6–12 months (Figures 3 and 4). In leave-one-out sensitivity analysis, exclusion of one study [23] reduced heterogeneity but increased pooled mortality to 16% (15–17%) (Figure 5). The funnel plot showed a slight asymmetry (Figure 6), while Egger’s test (*p* = 0.39) suggested no potential publication bias.

## Discussion

To our knowledge, this is the first meta-analysis to quantify long-term mortality after pediatric sepsis. Our pooled estimate of 11% (95% CI: 7–16%) post-discharge mortality highlights a clinically significant burden, with rates remaining broadly consistent across follow-up durations despite inter-study variability.

Long-term outcomes after hospital discharge remain an important but often underappreciated aspect of sepsis care. More than one-sixth of pediatric sepsis survivors are rehospitalized within 30 days, compared with about one in five among adult sepsis survivors [26,27]. In low-middle-income countries, nearly 50% of discharged children die within 6 months, with over half occurring outside healthcare facilities [28,29]. Reported post-acute mortality among adult sepsis survivors was 16.1%, higher than the pooled estimate for children in our analysis [30]. These findings collectively underscore persistent vulnerabilities among sepsis survivors, irrespective of age.

The drivers of post-discharge mortality in pediatric sepsis are multifactorial, including discharges against medical advice, residual underlying conditions, risky home environments, limited caregiver resources, and fragile healthcare infrastructure impeding follow-up care [29,31,32]. Mechanistic studies in adults reveal a persistent increase of inflammatory and immunosuppressive biomarkers correlating with late mortality [33], yet equivalent pediatric investigations remain scarce. The long-term physical, cognitive, and psychosocial sequelae in children have received less attention than in adults. Recent international guidance for adult sepsis increasingly emphasizes post-discharge assessment and follow-up, while such recommendations are notably lacking in pediatric sepsis guidelines [34]. Therefore, there is an urgent need to strengthen clinical care and follow‑up for pediatric sepsis survivors, aiming to reduce preventable post‑discharge deaths and other adverse outcomes.

This study is limited by several factors. First, limited studies precluded extensive subgroup analyses. Second, substantial heterogeneity inherent to proportional meta-analyses complicates generalizability [35]. Third, the absence of control cohorts restricts conclusions to associative observations, rather than causal inference or direct comparison to non-sepsis pediatric populations. Fourth, although funnel plot and Egger’s test showed no significant bias, its reliability is limited to fewer than 10 studies, and English-language restrictions may exclude relevant data, potentially leading to publication bias.

## Conclusion

This systematic review and meta-analysis identified an 11% long-term mortality rate following pediatric sepsis, underscoring the persistent risk of mortality after hospital discharge. High-quality longitudinal studies are urgently needed to determine modifiable risk factors and to inform improvements in evidence-based follow-up and individualized care.

## Supplementary Material

Supplementary material.docx

## Data Availability

The data supporting the findings of this study are included in this article and its supplementary material files. Further data are available from the corresponding author on request.
